# Unbiased analysis of knee cartilage thickness change over three years after sprifermin vs. placebo treatment – A post-hoc analysis from the phase 2B FORWARD study

**DOI:** 10.1016/j.ocarto.2024.100513

**Published:** 2024-08-23

**Authors:** Felix Eckstein, Susanne Maschek, Wolfgang Wirth, Christoph Ladel, Asger Reinstrup Bihlet, Chris Knight, Kenneth Somberg, Luping Zhao

**Affiliations:** a Research Program for Musculoskeletal Imaging, Center for Anatomy & Cell Biology, Paracelsus Medical University (PMU), Salzburg, Austria; b Ludwig Boltzmann Institute for Arthritis and Rehabilitation (LBIAR), Paracelsus Medical University (PMU), Salzburg, Austria; c Chondrometrics GmbH, Freilassing, Germany; d CHL4special Consulting, Darmstadt, Germany; e NBCD A/S, Copenhagen, Denmark; f Formation Bio, New York, NY, United States

**Keywords:** Post treatment efficacy, Clinical trial, DMOAD (sprifermin), Cartilage morphometry, Magnetic resonance imaging

## Abstract

**Objective:**

Post-treatment cartilage morphometry in the FORWARD study was performed without blinding to MRI acquisition order, involving potential reader bias. Here we obtained unbiased estimates of cartilage change post-treatment, reading year (Y)2 and Y5 MRIs with blinding to time point. We studied whether post-treatment cartilage thickness change differed between sprifermin- and placebo-treated knees.

**Methods:**

FORWARD was a 5-year randomized control trial in 549 knee osteoarthritis patients. Here, Y2/Y5 images were analyzed with blinding to relative temporal order and treatment group. Cartilage change during Y2→Y5 was obtained in 337 participants: n ​= ​57 treated with placebo intra-articular injections every 6 months (q6M); n ​= ​69 with 30 ​μg sprifermin every 12 months (q12 ​M), n ​= ​67 with 30 ​μg q6M, n ​= ​73 with 100 ​μg q12 ​M, and n ​= ​71 with 100 ​μg q6M between baseline (BL) and 18 ​M. Total femorotibial joint (TFTJ) cartilage thickness was the primary analytic focus.

**Results:**

TFTJ cartilage thickness change during Y2→Y5 was −26μm (SD64; 95%CI -32,-19) across the cohort; no statistically significant difference (p ​= ​0.80) was observed between Sprifermin treated or placebo arms (one-way ANOVA). All groups lost cartilage, but the treatment-related difference in cartilage thickness in Sprifermin arms relative to placebo at Y2 was maintained until Y5. Annualized cartilage change in placebo participants was −8.2 ​μm (SD21; 95%CI -14,-2.5) during Y2→Y5 vs. −5.4 ​μm (SD27; 95%CI -13,1.8) during BL→Y2; no significant difference was identified (*t*-test).

**Conclusion:**

FORWARD is the first study evaluating post-treatment benefits of a potential disease modifying osteoarthritis drug. Cartilage thickness gained with 100 ​μg sprifermin at Y2 is maintained to Y5 and thus appears viable and sustainable.

This is a post-hoc analysis of the FORWARD trial: ClinicalTrials.gov Identifier: NCT01919164.

## 1 Introduction

Sprifermin, or recombinant human fibroblast growth factor-18 (rhFGF18) has been shown to induce proliferation of hyaline cartilage-producing chondrocytes and to stimulate the synthesis of hyaline extracellular matrix in vitro and ex vivo [1], and to stimulate chondrogenesis and cartilage repair in a rat model of injury-induced osteoarthritis [2]. As a potentially anabolic intra-articular disease-modifying osteoarthriti drug (DMOAD), sprifermin has thus far been taken through extensive phase 1 and 2 programs [3]: In a first-in-human (FIH) study in 55 subjects eligible for knee replacement, sprifermin was applied over 6 months (M), with no relevant safety signals or systemic exposure seen [4]. A phase 2A proof-of-concept (POC) study in 192 participants revealed a statistically significant increase in cartilage thickness vs. placebo in knee osteoarthritis patients treated with the highest sprifermin dose (100 ​μg) in the total femorotibial joint (TFTJ) and lateral femorotibial compartment (LFTC) at the end of the 1-year study period; however, the primary structural endpoint, cartilage thickness change in the central medial femorotibial compartment, failed to meet statistical significance [5]. The participants showed a substantial improvement in pain during that trial, most so in placebo-treated patients [5]. The subsequent phase 2B study (FORWARD) included 549 participants with treatment applied up to 18 ​M, and revealed a statistically significant increase in cartilage thickness in the TFTJ over placebo at Y2 in the 100 ​μg cohorts [6]. Further, a doubling of the location-independent cartilage thickening score, and a substantial reduction of the cartilage thinning score were demonstrated in the 100 ​μg cohorts compared with a healthy reference cohort from the Osteoarthritis Initiative [7]. Although all FORWARD treatment arms displayed a substantial reduction in WOMAC knee pain, this decrease did not exceed that in the placebo group in any of the sprifermin-treated cohorts [6]. However, when restricting the analysis post-hoc to those participants with worse structural and symptomatic status at the beginning of the study (subcohort at risk ​= ​SAR), the anabolic benefit on femorotibial cartilage translated into a symptomatic benefit at Y3 and exceeded the minimally important clinical difference [8]. As FORWARD included a post-treatment observation period (Y2→Y5) that extended the study to 3.5 years after the last injection applied, it was shown that structural improvement and its translation to a symptomatic benefit in the SAR was maintained at Y5^9^. However, the cartilage thickness analyses following Y2^9^ were performed after the analysis between baseline (BL) and Y2 had been completed. While the former were accomplished “en bloc” and in one session, with the readers strictly blinded to the relative temporal order of MRIs acquired at BL, 6 ​M, 12 ​M, 18 ​M, and 24 ​M^6^, the analysis of the post-treatment time points (Y3, Y4 and Y5) was “added” piecewise later, as becoming available [9]. The previous BL→Y2 images were used as a reference and these measurements hence conducted without blinding to relative acquisition order [9]. Between-group comparisons could thus be made at Y3, 4 and 5, but the rates of change could not be computed without bias for the various dose- and placebo -groups over the post-treatment periods. As known from other musculoskeletal disease areas and recently shown for quantitative cartilage thickness measurement, unblinding readers to timepoint substantially affects the apparent longitudinal rate of cartilage thickness change [10]. The unbiased rate of change in cartilage thickness during the post treatment period (Y2→Y5) in FORWARD (or any other DMOAD trial), however, has not been previously determined, because of reader bias due to unblinding of post-treatment time points vs. baseline or time-points acquired during the treatment [9].

The objective of this study was to obtain unbiased rates of cartilage thickness change in the FORWARD cohort over the post treatment period (Y2→Y5). The analysis meant to elucidate whether the relative cartilage gain achieved during sprifermin treatment over the first two years was maintained over the post-treatment period up to Y5. Such data can provide clues as to whether the cartilage accumulated during anabolic treatment is structurally viable, mechanically competent and sustainable post treatment. To this end, Y2 images were re-read together with Y5, with blinding to acquisition order, to identify whether rates of cartilage change differ between Sprifermin-vs. placebo-treated knees.

## 2 Methods

FORWARD was a 5Y multicenter RCT [6,9], with the 549 knee OA patients randomized 1:1:1:1:1 to three once-weekly i.a. injections of placebo, 30 ​μg sprifermin every 12 (q12 ​M), or every 6 months (q6M), or 100 ​μg q12 ​M or q6M (last injection at 18 ​M). The study protocol was approved by independent ethics committees or institutional review boards at all study sites [6]; written informed consent was obtained from all participants, and the study was performed in accordance with the ethical principles of the Declaration of Helsinki [6,9].

**Fig. 1:**
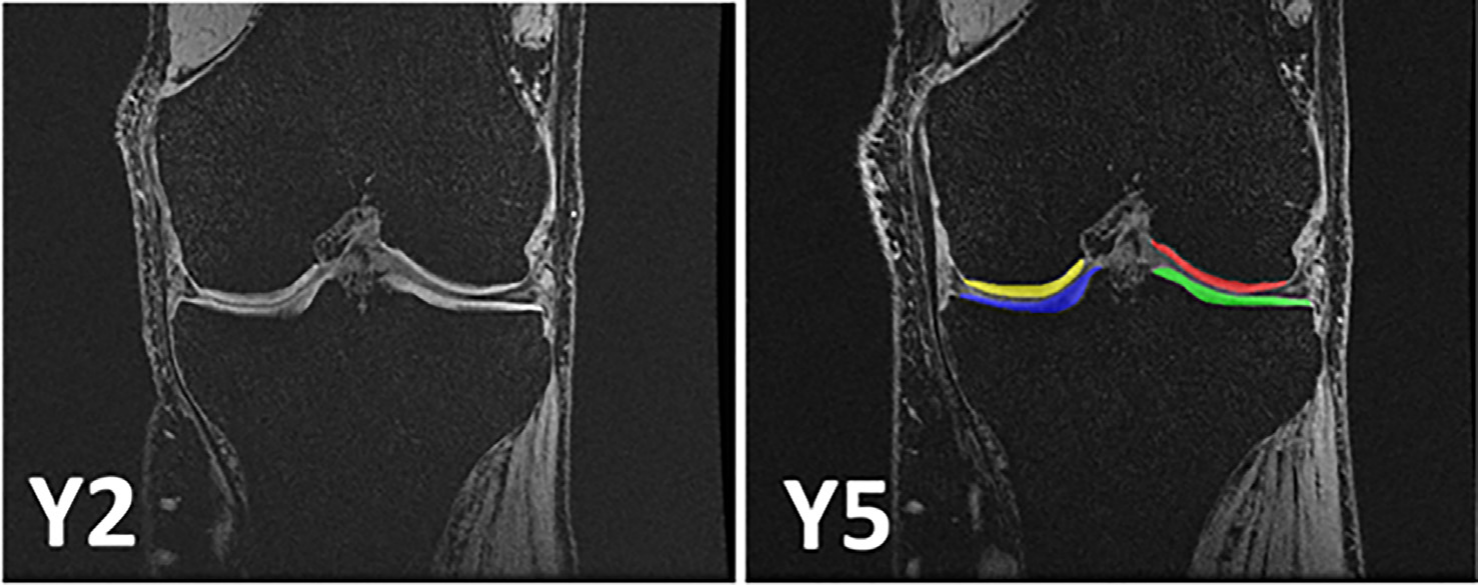
Coronal fast low angle shot (FLASHwe) MRI acquired over the post-treatent period of the FORWARD study at year 2 (Y2) and year 5 (Y5). The last sprifemin treatment (i.a. injection) was applied at 18 months (i.e. 6 months prior to Y2). Y2 image without segmentation and Y5 image with segmentation of the weight-bearing femorotibial joint cartilages. Blue ​= ​medial tibia; yellow ​= ​medial femur; ​= ​MFTC; Green ​= ​lateral tibia; red ​= ​lateral femur; ​= ​LFTC

After Y2, cartilage segmentation was accomplished on BL, 6, 12, 18, and 24 ​M MRIs by 7 experienced readers, each batch of 5 acquisitions processed by the same reader with blinding to acquisition order, using custom software (Chondrometrics GmbH 3.0)^3,6–9^. After Y5, Y2 and Y5 images (Fig. 1) were analyzed as pairs by the very same reader who segmented the baseline through Y2 data, and by the same software. In this case, the readers were blinded to relative temporal order of Y2 and Y5, and used already segmented BL MRI as a reference. The demographics of the participants studied over the treatment [6] and post-treatment periods [9] have been reported previously. As noted above, all MRIs of one patient were analyzed by the same reader across treatment and post-treatment time points. Importantly, readers were continuously blinded to “treatment group” throughout the study.

Blinded longitudinal cartilage thickness change during the post treatment period was obtained for 337 participants: n ​= ​57 treated with placebo; n ​= ​69 with 30 ​μg sprifermin q12 ​M, n ​= ​67 with 30 ​μg q6M, n ​= ​73 with 100 ​μg q12 ​M, and n ​= ​71 with 100 ​μg q6M over the prior BL→Y2 treatment period. These 337 subjects were those from the modified intent to treat (mITT) population who had complete cartilage MRI analysis at baseline, Y2 (both original and re-reading) and Y5 time points. The baseline characteristics were similar across the population studied in the current paper (n ​= ​337), the full mITT (n ​= ​494), and the difference between the full mITT and the cohort included here (n ​= ​157). The only small difference observed was that the 337 participants had a somewhat higher percentage (16%) of participants coming from Estonia and a lower one (6.8%) coming from Romania than those from the mITT who could not be included in the current analysis (n ​= ​157; 8.9% and 22%, respectively). As for the main study [6,9], TFTJ cartilage thickness was the primary analytic endpoint, but medial femorotibial compartment (MFTC) and LFTC cartilage thickness (Fig. 1), and location-independent cartilage thinning scores [7,11] were also evaluated. One-way ANOVA was used to test for any statistically significant differences between rates of cartilage thickness change of the 5 groups during the post treatment period. No adjustments on the baseline characteristics were made, because these were similar across the 5 treatment arms.

In order to assess whether the presence of a segmented (unblinded) BL MRI had an impact on the rates of cartilage thickness change observed between the blinded Y2 and Y5 images, when serving as a reference, the (annualized) rate of change was compared between the post-treatment period (Y2→Y5) and the treatment-period (BL→Y2) in the placebo cohort, using paired t-tests [6].

## 3 Results

**Fig. 2:**
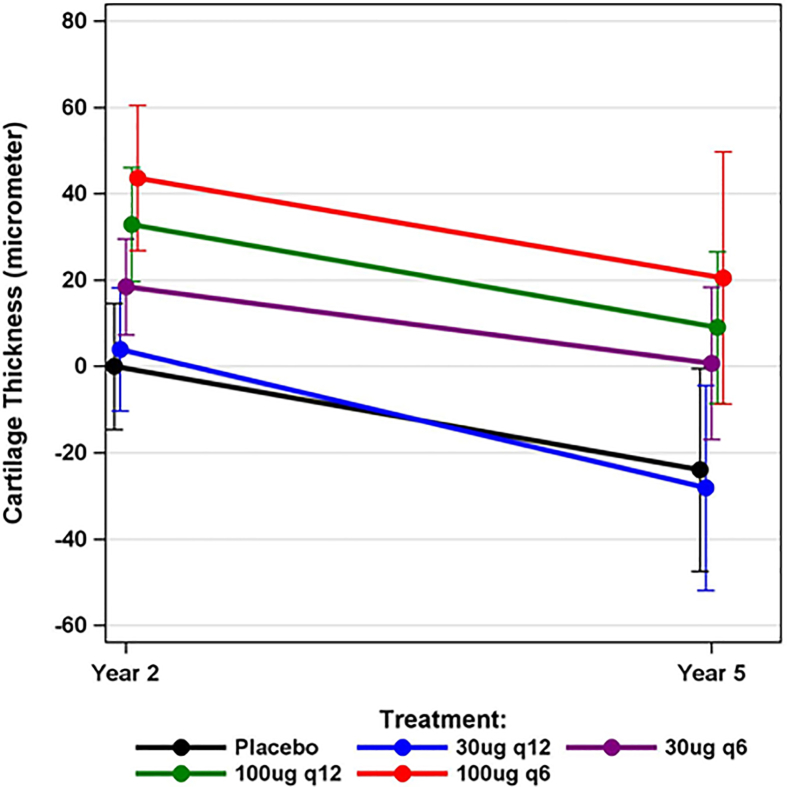
Change in total femorotibial joint (TFTJ) cartilage thickness between years 2 and 5 in the sprifermin- and placebo groups of the FORWARD trial (mean and 95% confidence interval). The lines are starting on the y-axis at year 2 ​at a value designating the gain in TFTJ cartilage thickness relative to placebo during the treatment period (baseline→year 2). No statistically significant difference in the cartilage thickness change was post treatment was identified between these groups, and the treatment benefit in cartilage thickness seen by sprifermin at year 2 was maintained at year 5.

The three-year change in TFTJ cartilage thickness during the Y2→Y5 post-treatment period across the entire cohort was −26μm (SD 64; 95% CI -32,-19); no statistically significant (or relevant) differences (p ​= ​0.80) were observed between any of the treatment or placebo arms (Table 1). The structural (cartilage thickness) treatment benefit observed in the 30μg/q6, 100μg/q12, and 100μg/q6 dose groups relative to placebo for TFTJ at Y2 was maintained between Y2 and Y5 (Fig. 2), with similar results in the MFTC (p ​= ​0.53) and LFTC (p ​= ​0.78) (Table 1). The location-independent thinning score (−0.88 ​mm; SD 1.18; 95% CI -1.01,-0.76) also did not differ significantly between the treatment and placebo groups (p ​= ​0.91; Table 1).

**Table 1:** Mean cartilage thickness change (± standard deviation) during the 3-year post-treatment period (Y2→Y5) of the FORWARD trial.

Group		Placebo	30 ​μg q12	30 ​μg q6	100 ​μg q12	100 ​μg q6
TFTJ	(μm)	−25 (±64)	−32 (±66)	−18 (±55)	−28 (±58)	−26 (±77)
MFTC	(μm)	−35 (±95)	−47 (±109)	−23 (±82)	−41 (±94)	−26 (±86)
LFTC	(μm)	−12 (±74)	−13 (±56)	−12 (±51)	−12 (±51)	−24 (±99)
Thin. Score	(mm)	−0.79 (±0.84)	−0.96 (±1.30)	−0.94 (±1.72)	−0.84 (±0.88)	−0.89 (±0.93)

TFTJ ​= ​total femorotibial joint; MFTC ​= ​medial femorotibial joint; LFTC ​= ​lateral femoro-tibial joint; Thin. Score ​= ​location-independent cartilage thinning score; no statistically significant difference was observed between groups (one way ANOVA) nor between the highest dose group (100 ​μg, q6) vs. placebo (*t*-test) in any of the cartilage metrics (FTJ, MFTC, LFTC, Thin. [= thinning] score).

The annualized TFTJ change in the placebo group during the post-treatment period (Y2→Y5) was −8.2 ​μm (SD 21; 95% CI -14, −3), and that during the treatment period (BL→Y2) was −5.4 ​μm (SD 27; 95% CI -13, 2). There as no significant difference between both periods (p ​= ​0.53). Values for the MFTC were −12μm (SD 32; 95% CI -20,-3) post-, and -6μm (SD 35; 95% CI -16,3) during treatment (p ​= ​0.35), and those for LFTC -4μm (SD 25; 95% CI -11,3) post-, and -4μm (SD 28; 95% CI -12,3) during treatment (p ​= ​0.99). The location independent cartilage thinning score between Y2 to Y5 amounted to −0.27 ​mm (SD 0.29; 95% CI -0.35, −0.20) post- and to −0.32 ​mm (SD 0.30; 95% CI -0.40,-0.24) during treatment in placebo participants, again without any statistical indication of a significant difference (p ​= ​0.31).

## 4 Discussion

The purpose of this study was, for the first time, to obtain an unbiased estimate of femorotibial cartilage thickness change post-treatment in FORWARD study participants, in whom the last treatment was applied at 18M. Y2 MRIs were re-read at the time of the Y5 analysis, with blinding to the relative image acquisition order. This was done to address whether post-treatment longitudinal cartilage thickness change differs between Sprifermin- and placebo-treated knees.

A limitation of the reading approach taken was that, albeit Y2 and Y5 MRIs were blinded to temporal sequence relative to each other, both were read together with already segmented baseline MRI as a reference, so that their order relative to baseline was obvious. This approach was necessary for the Y5 segmentations to be read in the same manner as the Y4 and Y3 MRIs previously, and to take a consistent approach in that respect [9]. However, to rule out a systematic bias on the rates of cartilage thickness change identified between blinded Y5 vs. Y2 by presence of unblinded BL analysis, we compared the annual rate of cartilage change of Y2→Y5 with that of BL→Y2 in the placebo group. These rates of change were found very similar and did not exhibit statistically significant differences in any of the femorotibial compartments. Presence of a segmented BL image (unblinded analysis) thus does not seem to have taken a relevant impact on the longitudinal (blinded) reading between Y2 and Y5. A further limitation was that no group was there in which treatment was maintained over 5 years, to explore the effect of continued Sprifermin therapy on cartilage.

Overall, the rates of change of the placebo and the treatmentment groups were relatively small. This is pertinent with the inclusion of only cases above a certain radiographic minimum joint space width (>2.5 mm medially) in FORWARD, resulting in participants with relatively mild or modest radiographic disease, most without baseline joint space narrowing, and hence relatively low rates of progression [6].

FORWARD is the first study to evaluate the longer-term benefit of a potential anabolic DMOAD on cartilage structure, for 3.5 years after the last injection [9], and to assess the sustainability of an anabolic effect on cartilage structure after treatment. This is a unique feature of this study, providing the opportunity to determine the longer lasting effect of the drug after the actual treatment has been terminated. Thus far most studies finished the measurements shortly after the end of the treatment period [3]. This limitation jeopardizes answering the question whether a structural benefit obtained by a certain intervention “survives” and is able to withstand the course of time. From the observations made here, we infer that the cartilage accumulated during anabolic sprifermin treatment appears to be structurally viable, mechanically competent, and sustainable, to withstand the mechanical enviroment post-treatment to the same extent as non-Sprifermin-treated osteoarthritic cartilage. This interpretation is in line with observations in the first in human study [4] that was suggestive of positive effects of Sprifermin treatment on mechanical properties of cartilage samples retrieved from the participants’ knees prior to knee replacement. Distinct molecular mechanisms have been identified that relate maintenance of cartilage to symptomatic outcomes (i.e. knee pain) [12,13]. Further, cartilage loss has been prospectively related to joint death (knee replacement) as the final clinical endpoint of knee OA [14,15]. Hence it is conceivable that maintenance of (anabolic) structural benefit is of great importance in achieving a relevant translation of structural (e.g. cartilage) benefit to symptomatic improvement.

In conclusion, the current study reveals that all sprifermin dose groups lost cartilage thickness throughout the post-treatment period, but not at greater rates than the placebo group. Hence, the structural benefit obtained by 100 ​μm i.a. Sprifermin at the end of the treatment period was maintained for at least three more years between Y2 and Y5.

## Author contribution

(1) All authors were involved in the conception and design of this review, or the selection of articles, or the analysis and interpretation of data in those articles.

(2) All authors contributed to drafting the article or revising it critically for important intellectual content. F.E. made the provision of the first complete draft of the article.

(3) All authors gave their final approval of the manuscript submitted.

## Role of the funding source

Funding for the MRI data analysis (cartilage morphometry) was provided by Merck KGaA as part of the FORWARD study analysis. Funding for the statistical analysis was provided by Formation Bio. No money was received for writing this article and no one other than the authors had any direct or indirect influence on the data presented.

F.E. takes responsibility for the work as a whole, from inception to the finished article.

L.Z. takes responsibility of the accuracy of the data and the statistical analysis.

## Declaration of competing interest

Felix Eckstein, Susanne Maschek, and Wolfgang Wirth are employees of Chondrometrics GmbH, a company that provides professional image analysis service to researchers in academia and to the pharmaceutical industry. They also are co-owners of Chondrometrics GmbH. They have received funding from multiple sources, including the pharmaceutical industry, Universities, and public national bodies (detailed list upon request).

Felix Eckstein has provided consulting services to Merck KGA, Tissue Gene, Galapagos, Novartis, 4P Pharma/4 Moving, and TrialSpark/Formation Bio.

Chris Ladel is a self-employed owner of CHL4special consulting. He has provided consulting services to Regenosine, Curnova, Charité hospital, TrialSpark/Formation Bio and ReumaNederland. He was formerly an employee of Merck KGaA, a company involved in the Sprifermin development program including the FORWARD trial.

Asger Reinstrup-Bihlet is a shareholder and employee of NBCD A/S. He was formerly an employee of Nordik Bioscience, a company involved in the FORWARD trial.

Chris Knight, Kenneth Somberg and Luping Zhao are shareholders and employees of Formation Bio, the company being the current owner of Sprifermin.
